# HNRNPA2B1 regulates the epithelial–mesenchymal transition in pancreatic cancer cells through the ERK/snail signalling pathway

**DOI:** 10.1186/s12935-016-0368-4

**Published:** 2017-01-10

**Authors:** Shengjie Dai, Jie Zhang, Shihao Huang, Bin Lou, Binbo Fang, Tingting Ye, Xince Huang, Bicheng Chen, Mengtao Zhou

**Affiliations:** 1 Department of Surgery, The First Affiliated Hospital, Wenzhou Medical University, 2 FuXue Lane, Wenzhou, 325000 Zhejiang Province People’s Republic of China; 2 Zhejiang Provincial Top Key Discipline in Surgery, Wenzhou Key Laboratory of Surgery, Wenzhou, Zhejiang Province People’s Republic of China

**Keywords:** Epithelial–mesenchymal transition, HNRNPA2B1, ERK/snail, Pancreatic cancer

## Abstract

**Background:**

Heterogeneous nuclear ribonucleoprotein A2B1 (HNRNPA2B1) is closely related to tumour occurrence and development, oncogene expression, apoptosis inhibition and invasion and metastasis capacities. However, its function in the epithelial–mesenchymal transition (EMT) of pancreatic cancer is not fully understood.

**Methods:**

By comparing various wild-type pancreatic cancer cell lines, we determined which have a higher expression level of HNRNPA2B1 accompanied by the higher expression of N-cadherin and vimentin and lower expression of E-cadherin. Therefore, to elucidate the role of HNRNPA2B1 in EMT, we generated models of HNRNPA2B1 knockdown and overexpression in different types of pancreatic cancer cell lines (MIA Paca-2, PANC-1 and Patu-8988) and examined changes in expression of EMT-related factors, including CDH1, CDH2, vimentin and snail.

**Results:**

The results show that HNRNPA2B1 promotes EMT development by down-regulating E-cadherin and up-regulating N-cadherin and vimentin, and also stimulates the invasion capacity and inhibits viability in human pancreatic cancer cell lines, the similar results in vivo experiments. Moreover, we found that HNRNPA2B1 likely regulates EMT progression in pancreatic carcinoma via the ERK/snail signalling pathway.

**Conclusions:**

The results of this work suggest that HNRNPA2B1 inhibition has potential antitumour effects, which warrants in-depth investigation.

## Background

Pancreatic carcinoma, which is nearly almost always fatal, is the fourth leading cause of cancer-related death worldwide, with fewer than 7% of patients surviving more than 5 years [1]. Chemotherapy (mainly based on gemcitabine and capecitabine) and radiation therapy remain the mainstay of treatment for pancreatic cancer, with poor efficacy in terms of extending patient survival [2, 3]. Additionally, although the curative effect is better, only 15% of patients who undergo surgery survive more than 5 years [4, 5]. In view of this dismal outcome, to enable treatment of this deadly disease with new druggable targets, there is an urgent need to thoroughly understand the molecular mechanisms of pancreatic cancer pathophysiology and development.

Recent studies have shown that heterogeneous nuclear ribonucleoproteins A2B1 (HNRNPA2B1), two structurally homologous proteins belonging to the hnRNP family, play important roles in normal development as well as in cancer processes in eukaryotic cells [6]. Indeed, elevated HNRNPA2B1 levels in tumours accelerate pre-mRNA processing via RNA binding, indicating the critical role of HNRNPA2B1 in the development of carcinoma. Recent work shows that the epithelial–mesenchymal transition (EMT), a process in which epithelial cells transform into cells with mesenchymal phenotypes, may be regulated by HNRNPA2B1. During EMT in tumour cells, up-regulation of vimentin and N-cadherin and down-regulation of E-cadherin (cell–cell adhesion molecules) promote cell invasion and metastasis in various cancers, including pancreatic cancer [7–10]. Other factors also directly or indirectly trigger EMT, including claudins, occludin, fibronectin, twist1, ZEB2 and snail [11–15]. Zhou et al. [16] indicated that HNRNPAB induces EMT and promotes metastasis of hepatocellular carcinoma by transcriptionally activating snail [16], and Barcelo et al. [17] showed that HNRNPA2B1 plays a key role in Kras-mutation associated pancreatic cancer [17]. Based on these findings, HNRNPA2B1 might play an important role in EMT during tumour development.

To date, however, no study has reported on the role of HNRNPA2B1 in EMT in pancreatic cancer. Thus, we aimed to determine the roles and potential application of HNRNPA2B1 in the EMT of pancreatic cancer by exploring the impact of HNRNPA2B1 knockdown and overexpression on EMT and the subsequent invasion and metastasis of pancreatic cancer cells. Furthermore, we here demonstrate the possibility that HNRNPA2B1 regulates and controls EMT in pancreatic cancer cells through the ERK/snail pathway. In conclusion, we conclude that HNRNPA2B1 plays a critical role in pancreatic carcinoma EMT.

## Methods

### Cell culture

The human pancreatic cancer cell lines used in this study include Patu-8988, MIA Paca-2 and PANC-1. The MIA Paca-2 and PANC-1 lines were obtained from the Institute of Biochemistry and Cell Biology, the Chinese Academy of Science (Shanghai, People’s Republic of China), and Patu-8988 cells were provided by Genechem (Shanghai, China). Patu-8988 cells were cultured in RPMI-1640 medium, and MIA Paca-2 and PANC-1 cells were cultured in DMEM. All media contained 10% foetal bovine serum, penicillin (100 U/mL), and streptomycin (100 μg/mL), and the cells were incubated at 37 °C with 5% CO_2_. Cell were harvested and passaged at approximately 80–100% confluence using phosphate-buffered saline (PBS) with 0.25% trypsin and 0.01% EDTA.

### Reagents and antibodies

Antibodies against HNRNPA2B1, E-cadherin, vimentin, N-cadherin, MMP7, MMP9, ERK1/2 and phosphorylated proteins were purchased from Abcam (Cambridge, USA). An antibody against β-actin was purchased from Bio-world Technology (St Louis Park, MN, USA). An antibody against snail was purchased from Cell Signaling Technology (Danvers, USA). Foetal bovine serum (FBS) was purchased from Sigma Chemical (St Louis, MO, USA). Roswell Park Memorial Institute (RPMI)-1640 medium, Dulbecco’s Modified Eagle’s Medium (DMEM), and trypsin were purchased from GIBCO (Grand Island, NY, USA). ERK-inhibitor (GSK2656157) was purchased from MedChem Express (New Jersey, USA) and gemcitabine was purchased from Jkchem (Shanghai, China).

### Knockdown and overexpression of HNRNPA2B1

A lentivirus plasmid containing a short hairpin RNA (shRNA) for HNRNPA2B1(KH)and its negative control (NKH) and a lentivirus plasmid containing HNRNPA2B1 driven by the CMV promoter (OH) and its negative control (NOH) were designed and produced by Genechem (Shanghai, China). For transfection, MIA Paca-2, PANC-1 and Patu-8988 cells were seeded in 6-well plates at 5 × 10^4^ cells and allowed to attach until nearly 30–40% confluent. After removal of the culture medium, the plates were washed three times with PBS, and lentivirus and 50 μg/mL polybrene was added to each plat. After 12 h of transfection, the transfection medium was replaced with standard medium. Cells were harvested for passaging or testing when they occupied 80% of the plate. All procedures were performed using biohazard safety equipment.

### Western blot analysis

Protein was extracted by adding RIPA buffer (Beyotime, Shanghai, China) with protease inhibitor (Beyotime, Shanghai, China) and phosphatase inhibitor (Roche Diagnostics GmbH, Mannheim, Germany). The final protein lysate was centrifuged, and the supernatant was collected. BCA (Beyotime, Shanghai, China) was used to measure the protein concentration. After denaturing, the protein mixture was separated using a 10% polyacrylamide gel; a prestained protein marker was included. The separated proteins were transferred to polyvinylidene difluoride (PVDF) membranes, which were blocked with 5% skim milk in Tris-buffered saline (10 mmol/L Tris–HCl, pH 8.0, containing 150 mmol/L NaCl and 0.1% Tween-20) for 2 h at room temperature before being incubated at 4 °C overnight with primary antibodies diluted in First Ab Dilute. The membranes were then incubated with horseradish peroxidase (HRP)-conjugated secondary antibodies (Bio-world Technology, MN, USA) for 2 h at room temperature after washing with TBST. The densities of the protein bands were recorded and measured by AlphaEaseFC (STandalone).

### RNA extraction and quantitative real-time PCR analysis

When the cells were approximately 90% confluent, total RNA was extracted using TRzol Reagent (Ambion, Carlsbad, California, USA) according to the manufacturer’s instructions. To obtain cDNA, reverse transcription was performed with RevertAid First Strand cDNA Synthesis Kit(Thermo, Manassas, USA). qRT-PCR was performed with SYBR Green Master (Biosystems, Foster City, CA, USA) using a 7500 Real-Time PCR System (Applied Biosystems, Foster City, USA). β-Actin was amplified as an internal standard, and ΔCt values were used for analysis of the quantitative real-time PCR data. Polymerase chain reaction (PCR) primers were purchased from Synbio Tech (Jiangsu, People’s Republic of China); the sequences are listed in Table 1.

**Table 1:** The sequences of the primers used for quantitative real-time PCR

Gene	Forward primer	Reverse primer
β-actin	GACATCCGCAAAGACCTG	GGAAGGTGGACAGCGAG
HNRNPA2B1	ATGGCTGCAAGACCTCATTC	TAATTCCGCCAACAAACAGC
E-cadherin	GACCGAGAGAGTTTCCCTAC	GGTGGGATTGAAGATCGGAG
N-cadherin	GTGACCGATAAGGATCAACC	TTGACCACGGTGACTAACCC
Vimentin	GGATGTTGACAATGCGTCTC	CTCCTGGATTTCCTCTTCGT
Mmp7	TGAGGATGAACGCTGGACG	CACTGCATTAGGATCAGAGGAA
Mmp9	AGTCCACCCTTGTGCTCTTC	GCCACCCGAGTGTAACCAT
ERK	ATCCCCATCACAAGAAGA	GCTTTGGAGTCAGCATTT
Snail	CTTCTCCTCTACTTCAGTCTCTTCC	TGAGGTATTCCTTGTTGCAGTATTT

### Cell viability assay

The different cell models were plated into 96-well plates containing 1 mL 10% FBS medium at a density of 10,000 cells per well and incubated for 24 h. The cells were starved in FBS-free medium for 12 h when the cells in each well reached 70% confluence. The medium was discarded, and the cells were washed once with PBS before; 100 μL FBS-free medium containing 10 μL Cell Counting Kit 8 (CCK8; Dojindo, Kuma-moto, Japan) solution was added to each well and incubated for 1–2 h. Cell viability is expressed as the fold change in absorbance at 490 nm for each well, as measured using a microplate reader (BioTek, Winooski, VT, USA).

### Cell invasion assay

Transwell Permeable Supports (Costar, Kennebunk, USA) with matrigel Basement Membrane Matrix (Corning, Franklin Lake, NJ, USA) bedding were used with an 8 μm porosity polyethylene terephthalate membrane. Cells (5 × 10^4^) were starved overnight and added to the upper chamber in 200 μL FBS-free medium; 500 μL RPMI-1640 (for Patu-8988 cells) or DMEM (for PANC-1 and MIA Paca-2 cells) with 10% FBS was added to the lower compartment as a chemoattractant. The cells were incubated for 24 h, and the filters were washed three times with PBS, fixed with 4% paraformaldehyde for 30 min, stained with 4 g/L crystal violet for 15 min and washed twice with PBS. The cells on the upper surface of the filter were gently scraped off. The stained cells in randomly selected fields were observed and counted using a 200× inverted microscope.

### Cell cycle analysis

The cell cycle analysis was measured using the Propidium iodide Kit for Flow Cytometry (Multiscience, Hangzhou, Zhejiang, China), according to the manufacturer’s instructions. After the treatments, cell cycle analysis was measured using a Thermo Attune and analyzed using the ModFit LT2.0 software (Coulter, Miami, FL).

### Establishment of the xenografted tumor model in nude mice

Athymic nude mice (BALB/C-nu/nu, 6–8 weeks old, female) were gained from the Animal Center of Chinese Academy of Science (Shanghai, China) and fed under specific pathogen-free conditions in the laboratory animal center of Wenzhou Medical University (Wenzhou, Zhejiang, China). We established a pancreatic xenografted tumor model through subcutaneous injection of 1 × 10^7^ pancreatic cancer cells per mouse. After the treatments, we observe the growth rate of tumor volume and the change of weight on each mice. All researches involving animals were approved by the Animal Ethics Committee of Wenzhou Medical University.

### Statistical analysis

Data are expressed as the mean ± standard error of the mean (SEM) for at least three independent experiments. Analysis of variance was utilised to distinguish differences in each group. A value of P < 0.05 was considered to be statistically significant. All analyses were performed using SPSS 16.0 software (IBM, USA).

## Results

### HNRNPA2B1 expression was associated with the mesenchymal phenotype in pancreatic cancer cell lines

**Fig. 1:**
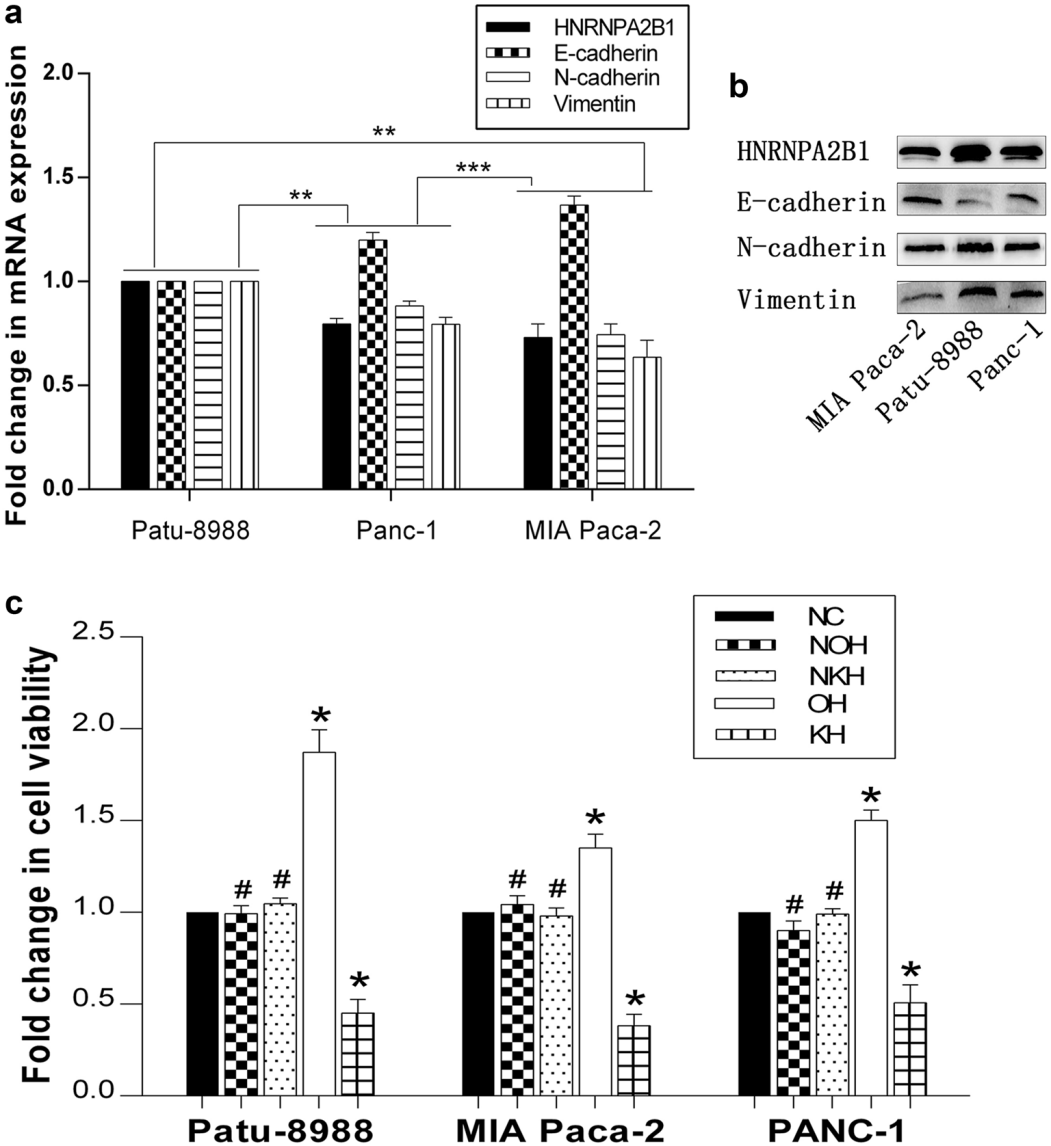
HNRNPA2B1 improves cell viability, and its expression was associated with EMT markers in pancreatic cancer cell lines. The mRNA expression levels of HNRNPA2B1 and EMT representative markers (E-cadherin and N-cadherin) in Panc-1 and Patu-8988 pancreatic cancer cells are presented as a histogram (**a**); HNRNPA2B1, E-cadherin, N-cadherin and vimentin were visualised by Western blotting (**b**); data represent the mean ± SEM, n = 3, *denotes P < 0.05 vs Patu-8988 groups. Fold change in cell viability of Patu-8988, PANC-1 and MIA Paca-2 cells (**c**); data represent the mean ± SEM, n = 3, *denotes P < 0.05 vs NC groups, **denotes P < 0.05 vs Patu-8988 cells, ***denotes P < 0.05 vs Panc-1 cells, ^#^means P > 0.05 vs NC groups

We first explored the relationship between HNRNPA2B1 and EMT in wild-type human pancreatic cancer cell lines (Patu-8988, MIA Paca-2 and PANC-1) by qRT-PCR and Western blotting and we found highest N-cadherin and HNRNPA2B1 expression and lowest E-cadherin in the Patu-8988 cell line compared to the Panc-1 and MIA Paca-2 cell line. The expression levels of HNRNPA2B1 in the different cells was directly correlated to the levels of N-cadherin and vimentin and inversely correlated to the levels of E-cadherin (P < 0.05; Fig. 1a, b).

### HNRNPA2B1 regulated cell viability and EMT in pancreatic cancer cell lines

**Fig. 2:**
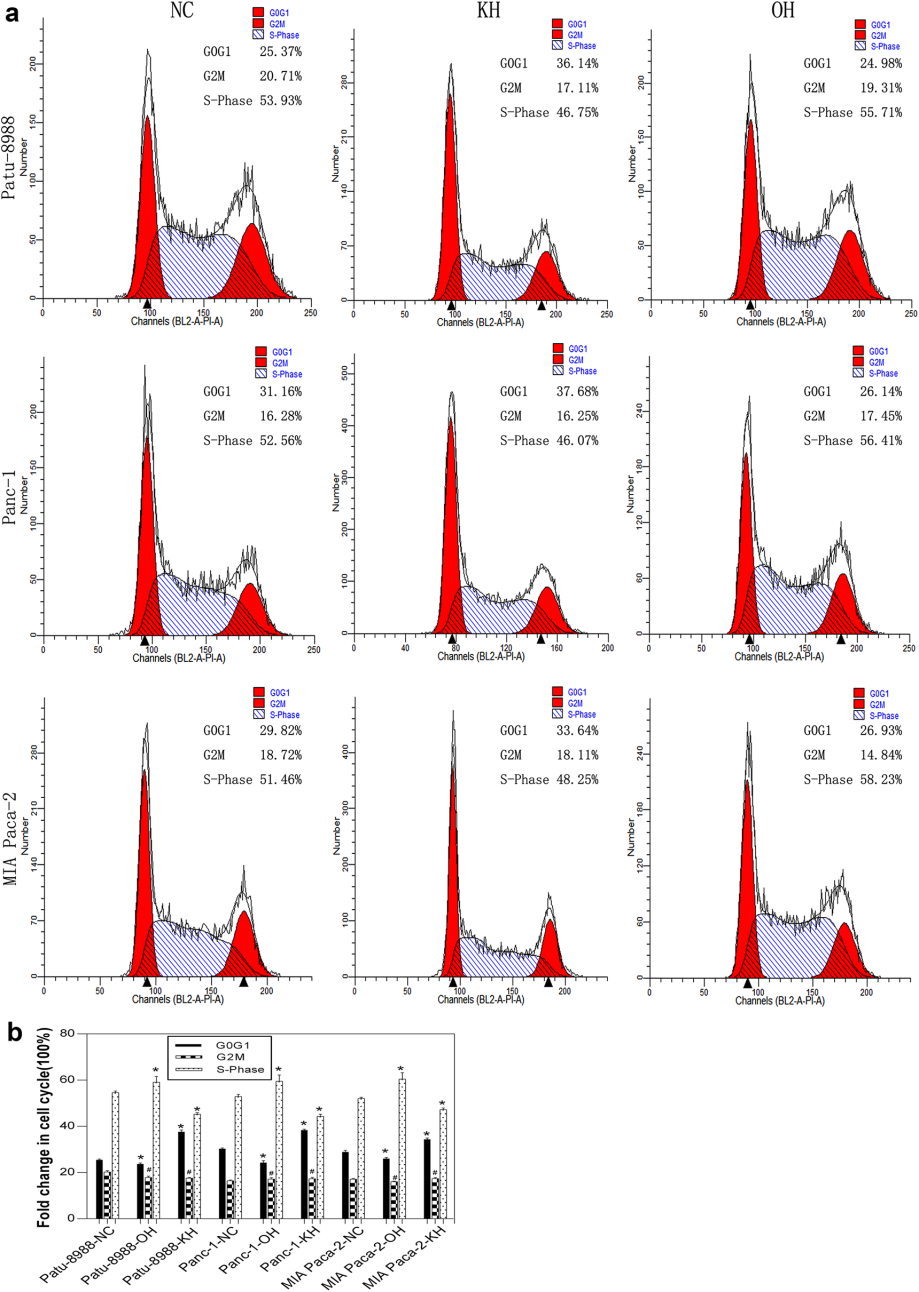
HNRNPA2B1 changes cell cycle in pancreatic cancer cell lines. The cell cycle of normal pancreatic cancer cell lines (Patu-8988, MIA Paca-2 and PANC-1) and its treated cells were visualised by flow cytometry (**a**). And a histogram of the proportion for cell cycle were drew in **b**. Data represent the mean ± SEM, n = 3, *denotes P < 0.05 vs NC groups, ^#^denotes P > 0.05 vs NC groups

We then generated cell models of HNRNPA2B1 knockdown and overexpression and examined accompanying changes in cell viability. All cell lines were treated with trypsinisation, and the cells in suspension were assayed using CCK8 after 24 h of incubation. Non-transfected pancreatic carcinoma cell lines were used as controls. The notable linearity of the graph revealed the lowest viability for the HNRNPA2B1 knockdown cells. Moreover, the non-transfected cells and the HNRNPA2B1 lentivirus negative control cells showed viability between the knockdown and overexpressing cells, with no statistical significance between them (P > 0.05; Fig. 1c). The level of HNRNPA2B1 expression was correlated with cell viability. To investigate if the cell cycle analysis is conforming to the consequence of CCK8 in the pancreatic cancer cell lines with HNRNPA2B1 knockdown or overexpression were generated by lentiviral gene delivery system. We were able to detect the G0G1 cell cycle arrest of Patu-8988-KH, Panc-1-KH and MIA Paca-2-KH cells and the S-Phase cell cycle increased of Patu-8988-OH, Panc-1-OH and MIA Paca-2-OH cells, but no significance difference was found in the G2M cell cycle of those cells (Fig. 2). So we concluded that HNRNPA2B1 could promote the proliferation of pancreatic cancer cells.

**Fig. 3:**
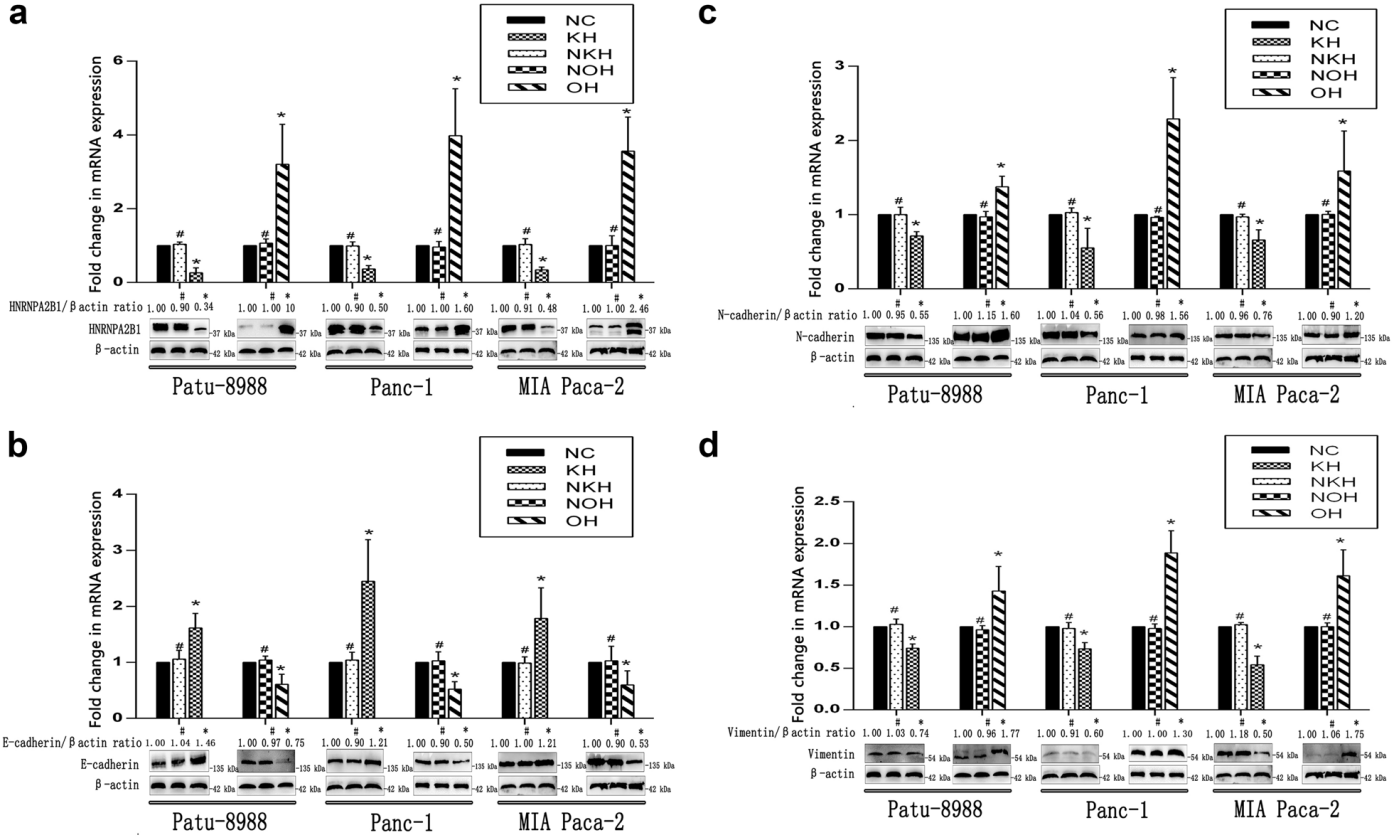
HNRNPA2B1 increases EMT in pancreatic cancer cell lines. HNRNPA2B1 (**a**), E-cadherin (**b**), N-cadherin (**c**) and vimentin (**d**) were visualised by qRT-PCR and Western blotting (histograms of mRNA expression on pictures of protein levels). β-Actin was used as an internal control. Data represent the mean ± SEM, n = 3, *denotes P < 0.05 vs NC groups, ^#^denotes P > 0.05 vs NC groups

We then sought insight into the relationship between HNRNPA2B1 and EMT. In addition to HNRNPA2B1, we also analysed EMT markers (E-cadherin, N-cadherin and vimentin). Western blotting assays showed that the protein levels of HNRNPA2B1 were decreased to 66% in Patu-8988, 52% in MIA Paca-2 and 50% in PANC-1 cells with knockdown but were increased to 1000% in Patu-8988, 246% in MIA Paca-2 and 160% in PANC-1 with overexpression. A similar pattern was observed by qRT-PCR (Fig. 3a). When HNRNPA2B1 was knocked down, the messenger RNA (mRNA) and protein levels of CDH1 (E-cadherin) increased, whereas these levels were decreased when HNRNPA2B1 was overexpressed (Fig. 3b). As expected, N-cadherin (Fig. 3c) and vimentin (Fig. 3d) showed trends opposite to that of E-cadherin. Furthermore, we confirmed that lower HNRNPA2B1 expression corresponded with higher E-cadherin expression and lower N-cadherin and vimentin expression in pancreatic cancer cells.

What’s more, we treated Patu-8988 cells with gemcitabine in 40, 80 and 160 µmol/L tentatively, and found the inhibiting effect of 160 μmol/L group is the strongest (Fig. 7a). Then, we respectively treated Patu-8988-OH, Panc-1-OH and MIA Paca-2-OH cells with gemcitabine in 160 μmol/L and discovered gemcitabine could inhibite EMT similar to HNRNPA2B1 (Fig. 7b, e). In conclusion, HNRNPA2B1 could regulated cell viability, EMT and the pharmacal sensitivity.

### HNRNPA2B1 promoted invasion in pancreatic carcinoma cell lines

**Fig. 4:**
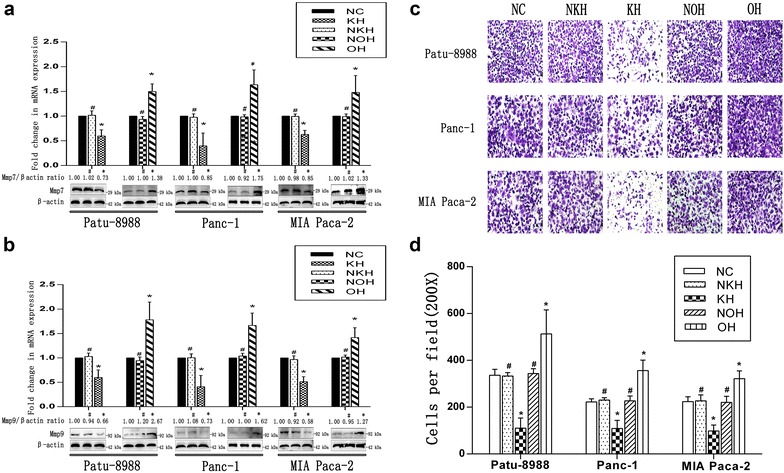
HNRNPA2B1 enhances cell invasion and expression of MMPs in pancreatic cancer cell lines. Mmp7 (**a**) and Mmp9 (**b**) were visualised by qRT-PCR and Western blotting (histograms of mRNA expression on pictures of protein levels). β-Actin was used as an internal control. The cell invasion ability **c** was evaluated in normal cells, cells transfected with a negative lentivirus control and cells transfected with a lentivirus. Cells were counted in 3 randomised 200× fields for invasion (**d**). Data represent the mean ± SEM, n = 3, *denotes P < 0.05 vs NC groups, ^#^denotes P > 0.05 vs NC groups

Because of the biological phenotype resulting from EMT, we evaluated the capacity of cells to invade to confirm whether HNRNPA2B1 is directly or indirectly associated with EMT. As shown by Fig. 4c and d, transwell invasion assays indicated a decrease in the invasion rate of KH cells but an increase in the invasion rate of OH cell. According to the results, HNRNPA2B1 could stimulate cell invasion after accelerating the development of EMT.

We also tested expression of MMPs under different conditions because MMPs enhance cell invasion by degrading the extracellular matrix. qRT-PCR and Western blotting showed significant decreases in the cellular levels of Mmp7 (Fig. 4a) and Mmp9 (Fig. 4b) after HNRNPA2B1 knockdown and increases after HNRNPA2B1 overexpression. These results are consistent with the invasion results.

**Fig. 5:**
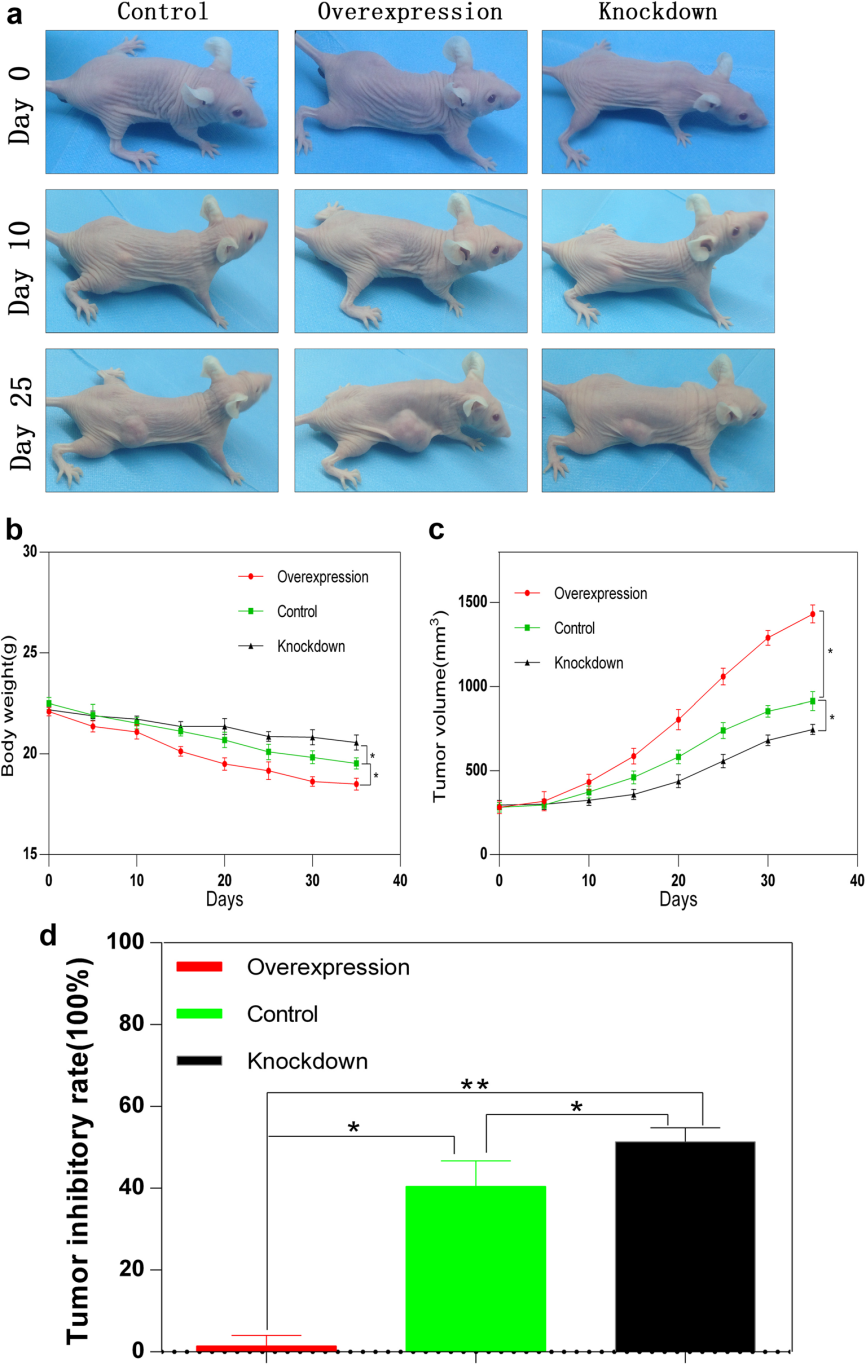
HNRNPA2B1 promotes the growth rate of xenografted tumor and decreases the weight in vivo. 1 × 10^7^ Panc-1, Panc-1-OH and Panc-1-KH cells were injected in subcutaneous tissues per athymic nude mouse. Mouse were put in the *blue drape* for photograph on day 0, day 10 and day 25 severally (**a**). The body weight (**b**) and tumor volume (**c**) of each mouse was measured and visualized as line graphs. Tumor inhibitory rate was calculated by using the Panc-1-OH group as a reference substance (**d**). Data represent the mean ± SEM, n = 3, *denotes P < 0.05 vs NC groups, **denotes P < 0.05 vs Panc-1-OH group

Furthermore, we established xenografted tumor in nude mouse via subcutaneous injection of 1 × 10^7^ PANC-1, PANC-1-KH and PANC-1-OH cells per mouse severally. As shown by Fig. 5a–c, we observed that the largest tumor volume and the thinnest in the PANC-1-OH-mice but an antipodal phenomenon in the PANC-1-KH-mice. Also, we found the tumor inhibitory rate of the PANC-1-KH group is the highest by using the PANC-1-OH group as a reference substance due to its highest expression of HNRNPA2B1 (Fig. 5d). These data are consistent with that HNRNPA2B1 could stimulate the growth and invasion in pancreatic cancer.

### HNRNPA2B1 regulated EMT progression via the ERK/snail pathway in pancreatic cancer cell lines

**Fig. 6:**
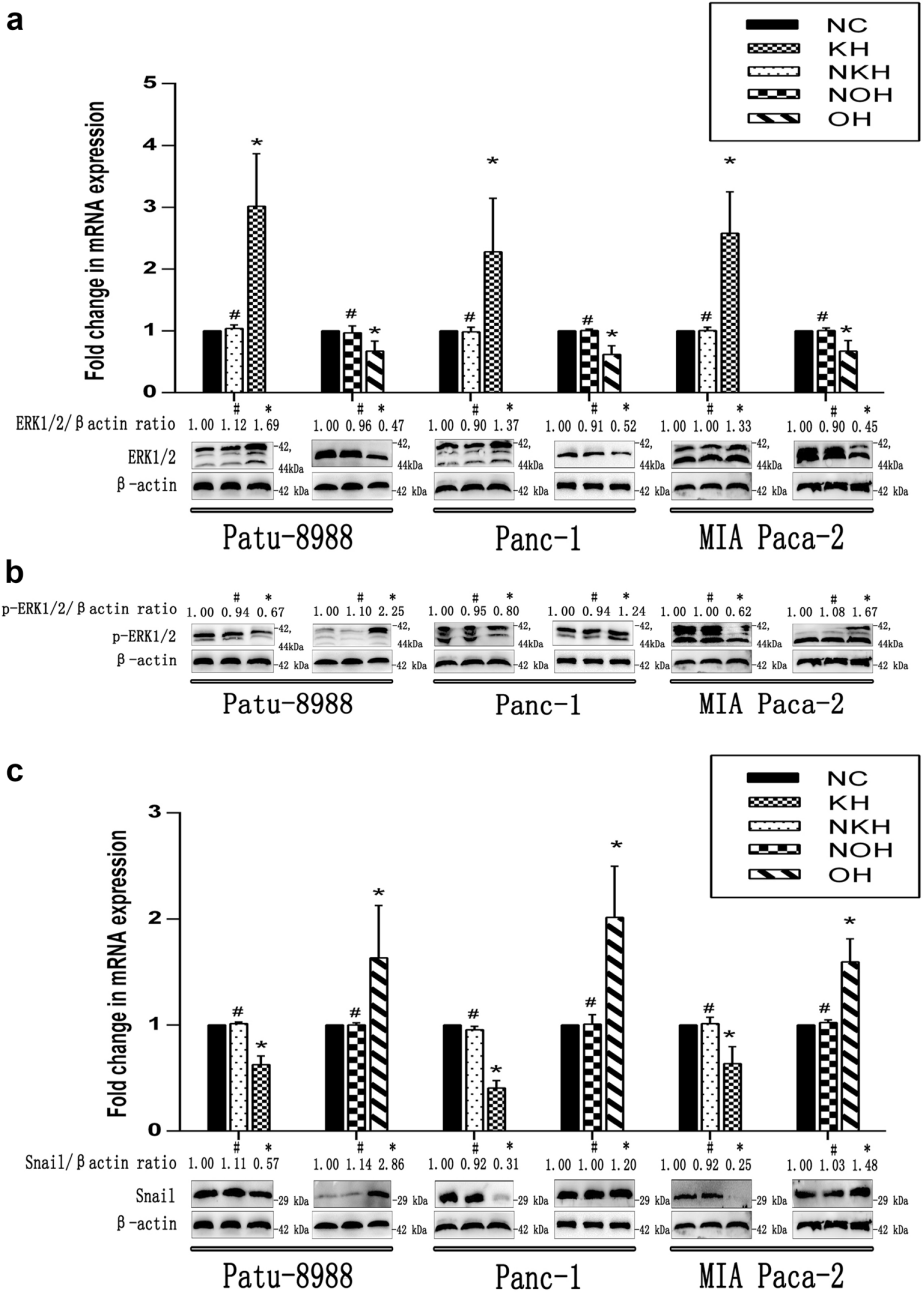
HNRNPA2B1promotes EMT via ERK/snail signalling in pancreatic cancer cell lines. Nonphosphorylated ERK1/2 (**a**), phospho-ERK1/2 (**b**) and snail (**c**) were visualised by qRT-PCR and Western blotting (histograms of mRNA expression on pictures of protein levels). β-Actin was used as an internal control. Data represent the mean ± SEM, n = 3, *denotes P < 0.05 vs NC groups, ^#^denotes P > 0.05 vs NC groups

**Fig. 7:**
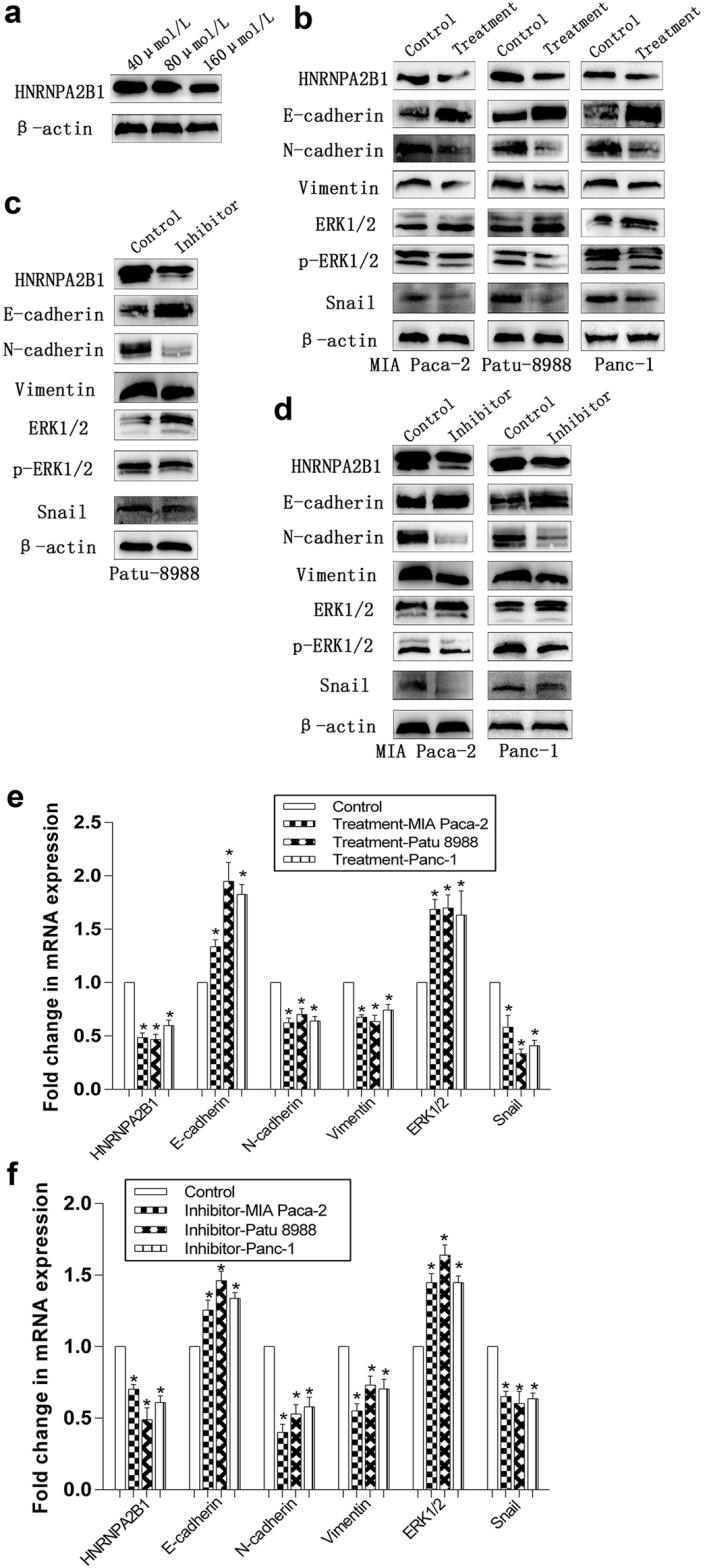
ERK inhibitor and gemcitabine inhibite HNRNPA2B1 and EMT in pancreatic cancer cell lines. The expression of HNRNPA2B1 in Patu-8988 cells treated with gemcitabine in 40, 80 and 160 μmol/L were visualised by Western blotting (**a**). The expression of HNRNPA2B1, E-cadherin, N-cadherin, Vimentin, ERK1/2, p-ERK1/2 and snail in Patu-8988-OH, Panc-1-OH and MIA Paca-2-OH cells treated with gemcitabine in 160 μmol/L were visualised by qRT-PCR (**e**) and Western blotting (**b**). The expression of HNRNPA2B1, E-cadherin, N-cadherin, Vimentin, ERK1/2, p-ERK1/2 and snail in Patu-8988-OH, Panc-1-OH and MIA Paca-2-OH cells treated with ERK inhibitor were visualised by qRT-PCR (**c**, **d**) and Western blotting (**f**). β-Actin was used as an internal control. Data represent the mean ± SEM, n = 3, *denotes P < 0.05 vs control groups

Splicing factor hnRNP A2 has been shown to activate the RAS-MAPK-ERK pathway by integrating A-RAF splicing signalling [18]. In addition, ERK activation markedly up-regulates expression of snail, one of the main transcription factors influencing EMT [19–25]. When HNRNPA2B1 was knocked down, snail expression was decreased according to both qRT-PCR and Western blotting (Fig. 6c), and that p-ERK1/2 expression was decreased according to Western blotting (Fig. 6b). In contrast, nonphosphorylated ERK1/2 remained at a higher level (Fig. 6a). The results obtained when HNRNPA2B1 was overexpressed were opposite to the results obtained when HNRNPA2B1 was knocked down. And then we respectively treated Patu-8988-OH, Panc-1-OH and MIA Paca-2-OH cells with ERK inhibitor, we observed that EMT was inhibited in the treated groups comparing with control groups (Fig. 7c, d, f). Therefore, we tentatively conclude that HNRNPA2B1 regulates EMT progression via the ERK/snail pathway in pancreatic cancer cell lines.

## Discussion

Pancreatic cancer, which currently has the worst prognosis, is the most common malignant tumour of the digestive system and among the top ten malignant tumours in our country [26]. Although surgery is the only radical treatment presently available, the curative rate of pancreatic cancer remains very low because surgery is often no longer an option at the time of diagnosis [27]. Accordingly, biotherapy treatments have become more popular over the past few years. Encouraging results have been reported by recent studies showing that pancreatic carcinoma could be inhibited by controlling several biomarkers such as SMAD4, CXCR2, ABCG2 and Kras [28–34]. In addition, Barcelo et al. showed that HNRNPA2B1 interacts with and regulates oncogenic Kras in pancreatic ductal adenocarcinoma cells, and Tauler et al. indicated that HNRNPA2B1 modulates EMT in lung cancer cell lines [17, 35]. However, a clear relationship between HNRNPA2B1 and EMT in pancreatic cancer and the signalling pathway involved remain elusive.

We previously discovered that the level of HNRNPA2B1 expression is higher in pancreatic cancer than in non-lesion tissue, as are other cancers, such as lung cancer [36], hepatocellular carcinoma [16] and glioblastoma [37]. Based on our study, we chose several pancreatic carcinoma cell lines to simulate different types of pancreatic cancers and generated models of HNRNPA2B1 depletion and overexpression using Crispr/Cas9 genetic technology. Unsurprisingly, conforming to the manifestation of an oncogene, both the mRNA and protein levels of HNRNPA2B1 were closely related with EMT, which plays an important role in the invasion and metastasis of cancer cells [38, 39]. Moreover, we found that the ERK/snail signalling pathway, regulated by HNRNPA2B1, plays a crucial role in pancreatic carcinoma.

EMT has a vital function in the process of phenotypic conversion [15, 40–42]. Accumulating experimental evidence suggests that many pancreatic cancer cell lines express higher levels of mesenchymal biomarkers, such as vimentin and N-cadherin, and lower levels of epithelial biomarkers, such as E-cadherin, than normal pancreatic cell lines. In our study, we found that the cell lines expressing higher levels of HNRNPA2B1 had enhanced invasive and migration capacities as well as lower expression of E-cadherin and higher expression of vimentin and N-cadherin than the others cells. Moreover, we detected levels of MMP7 and MMP9 expression and found that HNRNPA2B1 promoted expression of these MMPs, with a correlated change in of MMP expressions and EMT development. In addition, transwell invasiveness assays showed a greater number of cells passing through the membrane when MMP levels were higher due to extracellular matrix degradation. What’s more, vivo study showed that the cells with higher expression of HNRNPA2B1 could induce bigger xenografted tumor in mice. Therefore, we speculate that HNRNPA2B1 may be a potent inducer of EMT in pancreatic carcinoma. Meanwhile, snail, an important transcription factor up-regulation in EMT, is noteworthy because snail triggers EMT by coordinating the induction of mesenchymal biomarkers and the repression of epithelial biomarkers, which could induce EMT and promote metastasis of hepatocellular carcinoma. We found that the expression level of snail mRNA and protein consistently increased when HNRNPA2B1 was overexpressed. However, the concrete mechanism by which this occurs in pancreatic cancer requires further investigation.

As one of the most important intracellular pathways, RAS/RAF/MEK/ERK signalling causes multiple changes in the expression of various genes, transmitting signals from receptors to regulate gene expression and prevent apoptosis [43–46]. Recent studies have shown that activation of the ERK pathway, not only by growth factors but also by mutations occurring in cancer cells in the genes encoding RAS or RAF, contributes to EMT. RAS and RAF signalling also activate expression of snail1 and/or snail2, therefore promoting cell motility and invasive behaviour in cancer-associated EMT [13]. Moreover, Hsu et al. showed that snail promotes cell motility and invasive behaviour in cancer-associated EMT by activating the ERK signalling [22], HNRNPA2B1, a member of a heterogeneous group of nuclear ribonucleoproteins, has been shown to contribute to ERK activation by controlling A-RAF splicing [18]. We explored the relationship between snail and the ERK pathway and found that the expression level of snail was notably decreased with attenuation of ERK phosphorylation caused by HNRNPA2B1 knockdown. Futher more, when we inhibited the activation of ERK1/2, both of HNRNPA2B1 and EMT were decreased. Therefore, we tentatively conclude that HNRNPA2B1 promotes EMT by activating the ERK/snail pathway in pancreatic cancer, revealing a potential connection between HNRNPA2B1 and EMT. Nonetheless, the mechanism of specific subunit by which HNRNPA2B1 affects the intracellular RAS/RAF/MEK/ERK cascade is still unknown, and the mechanism by which HNRNPA2B1 transfers a signal to various pathways and which subunits are involved requires further investigation.

## Conclusion

In summary, our study shows that via ERK/snail pathway, HNRNPA2B1 might increase invasion ability by activating EMT phenotypes in pancreatic cancer. Our results not only provide a basis for establishing HNRNPA2B1-targeted molecular therapy in pancreatic cancer but also enrich the current understanding of HNRNPA2B1’s regulation of EMT and its potential signalling pathway.

## Abbreviations

EMT: epithelial–mesenchymal transition
NC: normal control
KH: cells transfected with HNRNPA2B1 shRNA lentivirus
NKH: cells transfected with HNRNPA2B1 knockdown negative control lentivirus
OH: cells transfected with HNRNPA2B1 overexpression lentivirus
NOH: cells transfected with HNRNPA2B1 overexpression negative control lentivirus
SEM: standard error of the mean
MMPs: matrix metalloproteinases
